# IT-assisted comprehensive geriatric assessment for residents in care homes: quasi-experimental longitudinal study

**DOI:** 10.1186/s12877-024-04824-6

**Published:** 2024-03-19

**Authors:** David Attwood, Jim Vafidis, James Boorer, Scarlett Long, Wendy Ellis, Michelle Earley, Jillian Denovan, Gerard ’t Hart, Maria Williams, Nicholas Burdett, Melissa Lemon, Suzy Hope

**Affiliations:** 1 Pathfields Medical Group, Plymouth, UK; 2 University of the West of England, Bristol, UK; 3 University of Exeter, Exeter, UK; 4 Royal Devon University Healthcare NHS Foundation Trust, Exeter, UK

**Keywords:** Advanced care planning, Frailty, Care homes, Primary care, Geriatric assessment

## Abstract

**Background:**

Frailty interventions such as Comprehensive Geriatric Assessment (CGA) can provide significant benefits for older adults living with frailty. However, incorporating such proactive interventions into primary care remains a challenge. We developed an IT-assisted CGA (i-CGA) process, which includes advance care planning (ACP). We assessed if, in older care home residents, particularly those with severe frailty, i-CGA could improve access to advance care planning discussions and reduce unplanned hospitalisations.

**Method:**

As a quality improvement project we progressively incorporated our i-CGA process into routine primary care for older care home residents, and used a quasi-experimental approach to assess its interim impact. Residents were assessed for frailty by General Practitioners. Proactive i-CGAs were completed, including consideration of traditional CGA domains, deprescribing and ACP discussions. Interim analysis was conducted at 1 year: documented completion, preferences and adherence to ACPs, unplanned hospital admissions, and mortality rates were compared for i-CGA and control (usual care) groups, 1-year post-i-CGA or post-frailty diagnosis respectively. Documented ACP preferences and place of death were compared using the Chi-Square Test. Unplanned hospital admissions and bed days were analysed using the Mann-Whitney U test. Survival was estimated using Kaplan-Meier survival curves.

**Results:**

At one year, the i-CGA group comprised 196 residents (severe frailty 111, 57%); the control group 100 (severe frailty 56, 56%). ACP was documented in 100% of the i-CGA group, vs. 72% of control group, *p* < 0.0001. 85% (94/111) of severely frail i-CGA residents preferred not to be hospitalised if they became acutely unwell. For those with severe frailty, mean unplanned admissions in the control (usual care) group increased from 0.87 (95% confidence interval ± 0.25) per person year alive to 2.05 ± 1.37, while in the i-CGA group they fell from 0.86 ± 0.24 to 0.68 ± 0.37, *p* = 0.22. Preferred place of death was largely adhered to in both groups, where documented. Of those with severe frailty, 55% (62/111) of the i-CGA group died, vs. 77% (43/56) of the control group, *p* = 0.0013.

**Conclusions:**

Proactive, community-based i-CGA can improve documentation of care home residents’ ACP preferences, and may reduce unplanned hospital admissions. In severely frail residents, a mortality reduction was seen in those who received an i-CGA.

**Supplementary Information:**

The online version contains supplementary material available at 10.1186/s12877-024-04824-6.

## Background

Frailty describes a state of reduced physiological reserve where seemingly trivial physiological stressors trigger dramatic deteriorations [1]. Interventions targeting frailty could improve health and quality of life, and reduce healthcare utilisation and cost [1]. Historically, frailty interventions have been performed by geriatricians as specialists in healthcare for older adults, but research by the Royal College of Physicians shows a severe shortage of geriatricians, with one geriatrician per 8031 people aged over 65 in England [2]. Approaches to managing frailty are therefore shifting to a population health strategy, with frailty assessment by General Practitioners (GPs) in primary care being recommended in numerous guidelines. Since 2017/18, frailty screening has also been incorporated in the General Practice General Medical Services contract in England for those over the age of 65, with the electronic Frailty Index (eFI) being suggested as a suitable tool to assist GPs to identify patients living with frailty [3]. Analysis from 2018/19 suggested that across England primary care practices, just 14.3% of eligible people were screened for frailty [3]. For those with severe frailty the General Practice Contract in England requires a clinical review incorporating an annual medication review, and discussion as to whether the person has fallen in the last 12 months (and onward referrals if needed). The aforementioned analysis found that even of those coded as severely frail, only 59.2% had a documented medications review, and just 3.7% a falls assessment [3]. Thus despite the guidelines, incorporating effective evaluation and, crucially, intervention for frailty into routine practice remains a challenge [2].

Another important component of good care for those with severe frailty, and thus likely to be nearing the end of their lives, is advance care planning (ACP) [4]. ACP is an intervention whereby people discuss their preferences for potential medical scenarios. In a UK care home study, of the 80% residents who chose to make an Advance Care Plan (ACP), 95% put their care home as their preferred place of death, and 94% did not want resuscitation [5]. Despite this, 2017 figures suggest 17.2% of NHS bed days are due to unplanned admissions in over-75s, and of all unplanned admissions in over-75s, 23.7% were for people in their last year of life [4].

Once in hospital, a well-established frailty intervention is the Comprehensive Geriatric Assessment (CGA): “*a multidimensional, multidisciplinary process which identifies medical, social and functional needs, and the development of an integrated/coordinated care plan to meet those needs*” [6]. However, particularly for those with severe frailty, admission to hospital is not always a beneficial experience [7, 8]. CGA has been shown to increase the likelihood of patients being alive and in their own homes at 12 months [9]. A recent trial demonstrated that compared to acute hospital admission, home-based CGA alongside Acute Hospital at Home care can also reduce the likelihood of people living in long-term residential care at 6 months [10].

Most available CGA evidence currently derives from these “reactive” CGAs, i.e. completed following acute decompensation. However it seems plausible that a more proactive and preventative community-based approach could yield dividends, potentially stopping, delaying or reversing frailty [9], with significant public health benefits. There is surprisingly little research into this thus far; indeed there are remarkably few published results from CGA in non-hospital settings, despite being identified as a top 10 research priority for older people with multiple conditions [11]. One of the few published studies investigating a proactive CGA approach in primary care involved 1604 individuals in Sweden. At 2 years, those who received a CGA had a 17% relative risk reduction in unplanned hospital admissions compared to usual care [12].

Although as a healthcare system we are increasingly starting to systematically assess people for frailty, further research also needs to be done to develop or assess effectiveness of interventions. “Reactive” CGAs have demonstrated an impact on institutionalisation rates, and “proactive” CGA may reduce unplanned hospitalisation rates. There is also evidence for the benefits of specific components of CGA; for example, medications reviews can reduce the risk of fall-related injuries for older people [13], and multifactorial falls risk assessments and management programmes can reduce the frequency of falls [14]. Community interventions have previously been shown to improve physical function of older people [10, 15], which should improve general health and resilience. Recognising those who might particularly benefit from such interventions, as well as ensuring medication reviews and falls assessments are completed, could be valuable both for individuals and at a population level.

We have previously developed and used our own Pathfields Tool to screen for and diagnose frailty clinically, including assessing the degree of frailty [16]. The Pathfields Tool uses risk factors such as age, dementia diagnosis and difficulty mobilising, as well as those scoring as having moderate or severe frailty as per the electronic frailty index (eFI) [17], to create a cohort of patients at high risk of having undiagnosed frailty. At the next interaction with a clinician, the system then prompts clinicians to assess the patient’s frailty, with 4 outcomes: not frail, mild frailty, moderate frailty, or severe frailty. Clinicians are guided by the Rockwood Clinical Frailty Scale [18] (CFS) and their knowledge of the patient and their functional status to make a diagnostic decision, though the result is a clinical diagnosis of no/mild/moderate/severe frailty rather than a CFS numerical score. In comparisons with the eFI, the Pathfields Tool identified more patients in a high-risk cohort, and a higher proportion of patients were subsequently diagnosed with frailty [16].

As part of ongoing quality improvement in our practice, we wanted to develop a proactive CGA process for those we had clinically diagnosed with frailty, incorporating both a CGA process, and ACP, particularly for those with severe frailty. We have recognised the importance of systematically incorporating such tools into the IT system, and thus have developed an IT-assisted CGA and ACP process which we have been progressively incorporating into routine care for our older patients diagnosed with frailty. Acknowledging that pre-expressed wishes are not always consistent, or may not always seem appropriate in acute scenarios, we investigated whether our proactive IT-assisted CGA and ACP process could increase adherence to expressed hospitalisation and place of death preferences, and reduce adverse health outcomes for care home residents, particularly those living with severe frailty. This is an interim analysis of early results, which we have presented as a quasi-experimental longitudinal study.

## Method

### Setting

Data was collected between 1st March 2019 and 30th April 2021 in residential care homes– i.e. homes that provide 24-hour personal care [19] and space for residents to socialise, but do not always have 24-hour registered nursing cover (and thus differ from “nursing homes” which have to have on-site 24-hour registered nursing cover). Residents are typically older adults who are unable to live independently at home, for example, due to cognitive or physical impairments. All permanent residents of older peoples’ residential care homes in a Devon Primary Care Network were included, as part of a locally commissioned service to improve residents’ care in care homes.

### IT-assisted comprehensive geriatric assessment and advance care planning

We developed an IT-assisted Comprehensive Geriatric Assessment (i-CGA), assisted by Target Health Solutions (THS, a company that enhances primary care IT). This was incorporated on our IT system, SystmOne, to offer proactive CGAs to residents.

Our i-CGA process encompasses standard CGA activities [1, 6] such as:

1. Holistic medical review (patient goals; long-term conditions review and optimisation; medication de-prescribing; advance care planning).
2. Assessment and optimisation of function (social situation; mood and cognition; activities of daily living; mobility and falls; nutrition, weight and swallow; continence; skin; hearing and vision).

The key difference from a “standard” CGA is that the i-CGA was designed for primary care professionals who work at pace, do not have the specialist knowledge of geriatricians or other similarly skilled professionals, and need to offer targeted, high-value interventions to potentially hundreds of patients on their frailty register.

To support this, our i-CGA tool offers the following:

1. **Rapid review** of previous CGA entries.
2. **IT-assisted deprescribing** of medications: on pushing a button, the IT system interrogates the patient’s medications, flagging “high-risk” drugs for review such as opiates, z-class drugs, and drugs with a high anti-cholinergic burden.
3. **IT-assisted CGA checklist**: the system prompts a clinician to complete all domains of CGA documentation.
4. **IT-assisted care planning**: unlike previous practice involving manually inputting care plans, the i-CGA selects and reconfigures the CGA entry into a care plan and an electronic Treatment and Escalation Plan (e-TEP). This is automatically shared with the patient, Out-Of-Hours medical services (GP and ambulance), acute, community and hospice providers. As well as improving speed and quality of CGA delivery, the information sharing in the i-CGA should improve adherence to care preferences.
5. **Population Health management**:

- **Ageing Well Dashboard**: THS software has enabled serial data extraction of near real-time read-coded data from the i-CGA, to populate an “Ageing Well” dashboard. This stratifies the older population by frailty and residential status, and allows monitoring of key performance metrics, such as whether resuscitation and hospitalisation preferences have been coded, and the prevalence of higher risk medication prescribing.
- **IT-assisted targeted reviews**: The Ageing Well dashboard enables clinicians to search the entire frailty register, highlighting for targeted review:

i. Patients on high-risk drugs such as opiates, z-class drugs, and drugs with a high anti-cholinergic burden.
ii. Patients with an incomplete CGA, or with a care plan or drug review that is more than a year old.

There were two practitioners involved in the study, both of whom contributed to patient care for both the usual care and i-CGA groups and received training in using the i-CGA tool before visiting care homes. Practitioners had a choice over whether to use the i-CGA tool, but decided to use the system due to its efficiency, particularly in end-of-life situations. Quality assessment was not needed, as the IT-assisted CGA was programmed to prompt clinicians if core domains were not completed, thus ensuring that a minimum standard of CGA was adhered to.

Other activities supported the i-CGA process, including an Older People’s “duty team,” consisting of an interdisciplinary team working on-call to respond specifically to older people’s urgent care needs (such as falls) and provide medical care in community, and an Older People’s admin team to ensure care plans were shared with Out-Of-Hours providers. From November 2019, the intervention was supported by an Ageing Well Multi-Disciplinary Team with local community services colleagues. The team included a GP, district nurse, community therapist, acute response practitioner and social care professionals, and the team coordinated the entirety of residents’ clinical care in the community by coordinating their CGA and continuing proactive case management as part of residents’ usual care.

#### Frailty assessment

In 2019 our Primary Care Network developed the Pathfields Tool [16, 20, 21]. This facilitates systematic frailty assessment and clinical diagnoses by General Practitioners (GPs), who use the Rockwood Clinical Frailty Scale [18] supplemented by clinical and longitudinal knowledge of the patient to frame a diagnosis of no/mild/moderate/severe frailty. All participants received frailty assessments using this Pathfields Tool [16].

### Analysis

Two groups were followed up for a year:

- **Intervention group (i-CGA)**: Follow-up commenced on i-CGA completion.
- **Control group (usual care)**: Follow-up commenced on frailty diagnosis.

As CGA is a recommended intervention for frailty, there was a concerted effort by the primary care network to offer all residents the opportunity of receiving an i-CGA. Residents were not randomised to groups, but were instead initially prioritised based on clinical need; i-CGA was offered first to residents identified by care home staff who were approaching end-of-life, e.g. who were showing signs of rapid physiological deterioration or were receiving late-stage care, or were experiencing a physiological catastrophe that required urgent medical care. From March 2020, i-CGAs were then completed proactively through regular dedicated care home sessions as part of weekly ‘home rounds’.

Primary outcomes included:

- Proportion of residents who had completed an ACP.
- Unplanned hospital admissions and bed days/person year alive.
- Mortality.
- Place of death.

### Data sharing and ethics

As this was a quality improvement activity evaluating direct patient care [16], data was evaluated with a linked dataset and an information sharing agreement between the local hospital and GP surgery. General Data Protection Regulations articles 6 and 9 offer a lawful basis for this. A Data Protection Impact Assessment was completed to mitigate against data-sharing risks. All patient identifiable details were removed apart from NHS number, frailty diagnosis, age, dementia diagnosis, i-CGA completion, ACP preferences, date, and place of death. This register was shared with the Community Services Business Intelligence unit, which extracted hospitalisation metrics and location of death.

### Statistical analysis

Data was analysed using the statistics programme R [22]. Baseline participant characteristics are presented as means or medians depending on whether they were normally distributed, and were analysed using Student t-test/Mann-Whitney U test for continuous variables, and χ2/Fisher’s exact test for categorical variables.

The proportions of residents with documented ACP discussions, and who achieved their preferences, were analysed using the Chi-square test of association. For unplanned hospital admissions and bed days, baseline data for the i-CGA group was taken for the year preceding i-CGA completion, and for the control group, the year preceding frailty diagnosis. Follow-up data was taken for the year following these points. The outcomes included total numbers of unplanned hospital admissions/person years alive and unplanned bed days/person years alive. Numbers per person year alive were used given the high mortality. The formula used was:

Total number of admissions or bed days/total number of days alive x 365.

The control and i-CGA groups’ rates were compared using the Mann-Whitney U test. Survival at one year was estimated using Kaplan–Meier survival curves within R’s ‘survival’ package [23].

## Results

Results overall, and by frailty severity are shown in the Tables and Graphs; however, this analysis focused on the results from those with severe frailty. All residents were assessed as having frailty of some degree. At evaluation, 296 residents had completed follow-up, with 100 in the control group and 196 in the i-CGA group. Baseline characteristics in Table 1 show no difference between groups in frailty severity or dementia prevalence. The follow-up year started pre-pandemic for 97% of the control group and 45% of the intervention group.

**Table 1:** Baseline characteristics of residents in care homes

Demographics	Total (*n* = 296)		Control (*n* = 100)		i-CGA (*n* = 196)		P-value
**Frailty status**							
Mild frailty, n (%)	29	(10)	13	(13)	16	(8)	0.28
Moderate frailty, n (%)	100	(34)	31	(31)	69	(35)	0.62
Severe frailty, n (%)	167	(56)	56	(56)	111	(57)	0.93
**Dementia diagnosis**, n (%)	170	(57)	53	(53)	117	(60)	0.51
**Female**, n (%)	213	(72)	58	(58)	155	(79)	0.073
**Age**, mean (SD)	88.7	(6.97)	87.3	(6.9)	89.4	(6.9)	**0.015**
**Follow-up period start date**							
Pre-pandemic (before 17/3/20)	186	63%	97	97%	89	45%	**< 0.0001**
During Pandemic (after 17/3/20)	110	37%	3	3%	107	55%	**< 0.0001**

Table 2 shows a summary of outcomes for the i-CGA and control groups at follow-up, both overall and for residents living with severe frailty. Outcomes included having documented ACPs with hospitalisation and resuscitation decisions, changes in unplanned hospital admissions/person year alive and bed days/person year alive, mortality, and place of death.

**Table 2:** Outcomes after 1 year for i-CGA and control groups, for all residents and those living with severe frailty

Outcomes	Frailty Status	Total	Intervention group		Treatment difference	P value
			Control (*n* = 100)	i-CGA (*n* = 196)		
**Change in unplanned admissions / person years alive**	Overall	0.4 ± 0.36	1.01 ± 0.88	0.04 ± 0.34	-0.97 ± 0.94	0.16
	Severe frailty	0.29 ± 0.56	1.18 ± 1.46	-0.16 ± 0.10	-1.34 ± 1.52	0.22
**Change in unplanned bed days / person years alive**	Overall	3.9 ± 3.8	8.4 ± 8.0	1.7 ± 4.1	-6.8 ± 9.0	0.45
	Severe frailty	1.5 ± 5.0	4.6 ± 9.6	0.04 ± 5.9	-4.5 ± 11.2	0.99
**Advance care plan in place (%)**	Overall	91	72	100		**< 0.0001**
	Severe frailty	90	71	100		**< 0.0001**
**Mortality (%)**	Overall	51	57	48		0.1
	Severe frailty	62	77	55		**0.0013**
**Died in care home (%)**	Overall	74	68	78		0.41
**Died in hospital (%)**		19	23	16		0.26
**Died in unknown location (%)**		7	9	6		0.43
**Died in care home (%)**	Severe frailty	79	72	84		0.337
**Died in hospital (%)**		13	21	7		**0.008**
**Died in unknown location (%)**		9	7	10		0.467

Raw data overall, and by frailty severity, is shown in Supplementary Table 1. All i-CGA residents (100%) had documented ACPs outlining resuscitation and hospitalisation preferences, compared to 72/100 in the control group with documented resuscitation decisions (*p* < 0.0001), and 71/100 with hospitalisation preferences (*p* < 0.0001). In both groups, where documented, 97% preferred to “allow a natural death” (191/196, 70/72) when discussing resuscitation preferences. Of those with severe frailty in the i-CGA group, 85% (94/111) expressed a preference not to be hospitalised in the event of becoming unwell. Where documented in the severe frailty control group, 62.5% (25/40) preferred not to be hospitalised.

**Fig. 1:**
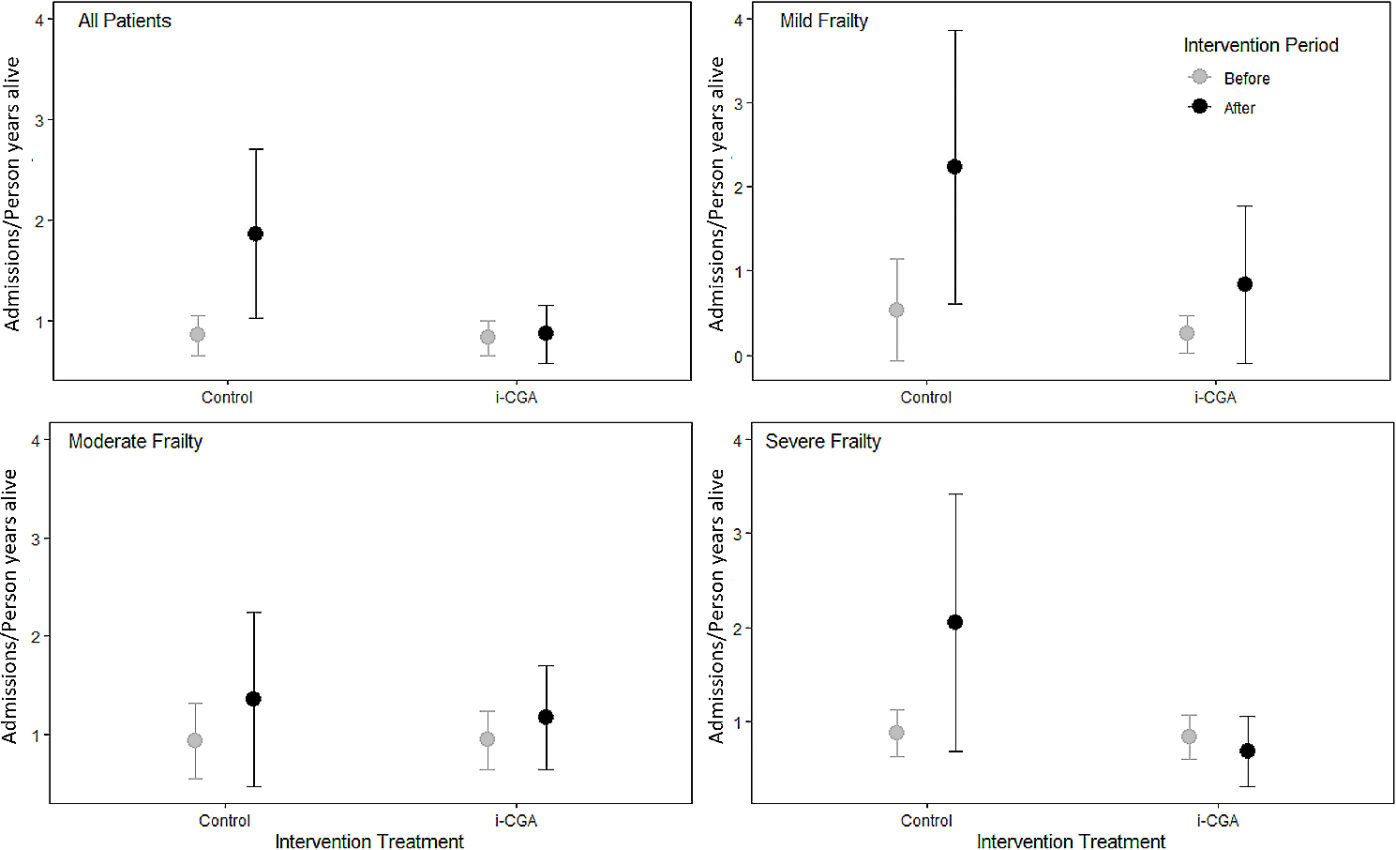
Mean baseline and follow-up hospital admissions/person year alive in control and intervention groups

Figure 1 shows the total number of unplanned hospital admissions/person year alive before and after follow-up (raw data overall, and by frailty severity, in Supplementary Table 2). In the control group, the overall number of unplanned hospital admissions/person year alive was 0.85 (95% confidence interval ± 0.20) in the year preceding diagnosis, increasing to 1.86 ± 0.84 the following year. In the i-CGA group, admissions remained similar: 0.83 ± 0.17 in the year preceding intervention, increasing to 0.87 ± 0.29 the following year. This gave an overall treatment difference − 0.97 ± 0.94 admissions/person year alive, *W* = 10,778, *p* = 0.16, favouring the i-CGA group.

For those with severe frailty in the control group, the total number of unplanned hospital admissions/person year alive was 0.87 ± 0.25 in the year preceding diagnosis, increasing to 2.05 ± 1.37 the following year. In the i-CGA group, admissions fell, from 0.84 ± 0.24 in the year preceding intervention, to 0.68 ± 0.37 the following year. This gave a treatment difference of -1.34 admissions/person year alive with 95% confidence intervals ± 1.52 (*W* = 3462.5, *p =* 0.22).

The mean number of unplanned hospital bed days/person year alive in the control group increased substantially from baseline to follow-up, from 8.8 ± 2.9 to 17.2 ± 8.1 (shown in Table 2 and Supplementary Fig. 1). In the i-CGA group, the number increased slightly, from a baseline mean of 7.1 ± 1.6, to 8.7 ± 4.0 at follow-up. This gave an overall treatment difference of -6.8 ± 9.0 bed days, *W* = 3420.4, *p* = 0.45 (Table 2), favouring the i-CGA group. For those with severe frailty, the mean number of unplanned hospital bed days/person year alive in the control group increased from 10.1 ± 4.2 to 14.6 ± 9.4. In the i-CGA group, the mean stayed the same, from 7.1 ± 2.2 to 7.1 ± 5.4. This gave a treatment difference of -4.5 ± 11.2 bed days, *p* = 0.99.

**Fig. 2:**
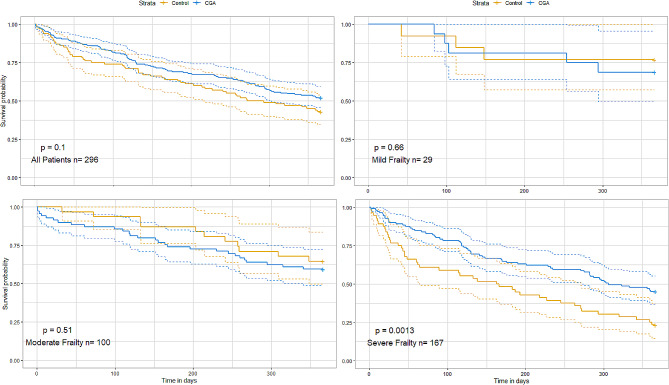
Kaplan-Meier survival curves over one year for i-CGA and control groups

At one year, overall, 151/296 (51%) residents had died: 57% of the control group (57/100), and 48% (94/196) of the i-CGA group, *p* = 0.1. Figure 2 shows Kaplan-Meier survival analyses for the follow-up year for both groups. There was a significant difference in one-year mortality in those with severe frailty: 77% (43/56) of the control group died in the follow-up year, compared to 55% (62/111) of the i-CGA group, *p* = 0.0013.

68% (39/57) of control residents died in their care home, compared with 78% (73/94) of i-CGA residents. Where documented, end-of-life preferences were largely adhered to. In the control group, where ACP had been discussed, 25/40 (63%) preferred not to be hospitalised. 21 (84%) of these died, 18/21 (86%) in their care home. In the i-CGA group, 94/111 (85%) preferred not to be hospitalised. 52 (55%) died, 47/52 (90%) in their care home.

15/40 (38%) of the control group with severe frailty had a documented preference to be hospitalised. 6 (40%) of these died in the follow-up year, 3–4 (50–67%) in hospital (missing data for one). In the i-CGA group 17/111 (15%) had expressed a wish to be hospitalised. 9 (53%) died, 3–5 (33–56%) in hospital (missing data for 2). Of the 16/56 (29%) control group residents with severe frailty but no documented hospitalisation preferences, all 16 (100%) died, 4–5 (25–31%) in hospital.

## Discussion

### Summary

The proportion of residents of care homes with documented ACP preferences was significantly higher in the i-CGA group compared to the control group. Where documented, adherence to preferences was high. For those with severe frailty, one-year mortality was significantly lower in the i-CGA than control group. No significant difference was demonstrated between unplanned hospitalisation rates between the intervention and control groups, though arguably there was a trend in the follow-up year to increased hospitalisation rates in the control group, in contrast to remaining static in the intervention group.

### Strengths and limitations

A key strength of the i-CGA process was sharing ACPs with the resident and local healthcare providers. Communicating the care plan increases residents’ chances of dying in their preferred place of care, and may affect hospitalisation rates. In residents with severe frailty, there was a large discrepancy between the documented hospitalisation preferences of the i-CGA and control groups. This disparity may be accounted for by some control group ACP discussions having occurred long before the study onset, meaning that documented preferences may not have been up-to-date and concurrent with their recently confirmed frailty diagnosis. If this is the case, it would suggest that a higher proportion of control group residents with severe frailty may have liked to have been admitted to hospital in the event of an acute deterioration in health, supporting the importance of accurate ACP documentation in this population. There may have also been a pandemic effect on expressed preferences, as discussed below.

Interestingly, there was a non-significant mortality *in*crease seen in i-CGA residents (compared to controls) living with mild/moderate frailty. Just eight residents with mild frailty died in total, and the small sample may well be misleading. For those with moderate frailty, raw data analysis showed 7 i-CGA residents died within 28 days of receiving an i-CGA, compared with no control deaths within 28 days. This may have been partly as in the initial stages of the project, the i-CGA process was done according to clinical need, given it was found to be helpful for end-of-life scenarios (with a focus on ACP), i.e. introducing a mortality bias. THS have since updated their software to differentiate “proactive” from “end-of-life scenario” i-CGAs. In addition, compared to the control group, more people in the i-CGA group were followed up during the coronavirus disease (COVID-19) pandemic, and unfortunately mortality in care homes was disproportionately higher during the pandemic [24].

Unplanned hospitalisation rates were lower in the i-CGA group. This may simply reflect adherence to residents’ documented preferences, as i-CGA residents were more likely than control residents to prefer not to be hospitalised. A confounding factor was the COVID-19 pandemic, with 97% of the control and only 45% of the i-CGA group starting follow-up pre-pandemic. The pandemic may have affected residents’ preferences, e.g. due to the media campaign to reduce admissions to “protect the NHS”, or because of fearing catching COVID-19 in hospital. Dementia rates were 57%, but documented preferences typically result from discussions between residents, family members, care home staff and GPs, so conversations were likely to have been affected by the pandemic.

In this study, although 97% of the control group and only 45% of the i-CGA group were followed up pre-pandemic, mortality for those with severe frailty was significantly lower in the i-CGA than control group. The proportion of those with severe frailty who died in hospital was also significantly reduced for the i-CGA group compared to the control group. The overall mortality difference between the two groups suggests that mortality was reduced for those with severe frailty, rather than reflecting a change in the distribution of location of mortality. The reduced mortality seems relevant as possible evidence of an early i-CGA benefit, or further evidence that for those with severe frailty, being admitted to hospital may not always be advantageous [7, 8].

Potential bias may have occurred from the opportunistic (non-randomised) approach to completing the intervention. Instead of being randomly allocated, residents were initially prioritised to receive i-CGA based on their clinical need, where residents who were receiving end-of-life care or who were showing rapid physiological deterioration received the i-CGA intervention first. This may actually strengthen the findings of an i-CGA benefit, as residents who underwent i-CGAs earlier were more likely to have been unwell than residents who received usual care. Furthermore, Table 1 does not show evidence of a selection bias, as there were no significant differences in frailty severity or dementia between the i-CGA and control groups. From March 2020, i-CGAs were then provided in regular ‘home rounds’ as part of the Enhanced Health in Care Homes service requirements. Not all residents and not all care homes had received the i-CGA intervention by the time of this interim analysis, thus allowing for a quasi-experimental analysis.

Finally, one limitation is that it is unclear whether the mortality differences are a result of receiving CGA and/or of having ACP preferences documented. As the focus of this quality improvement study was on implementing IT-assisted CGA (which included ACP) for primary care practitioners and comparing its effectiveness to usual care, this question may be better addressed in a larger study with more participants and without the potential confound of the pandemic. Since CGA is a holistic intervention that includes ACP discussions, systematically implementing CGA would confer not only any benefits of ACP discussions, but also the benefits of other components of CGA.

### Comparison with existing literature

Previous research demonstrated that proactive community-based CGAs reduced unplanned hospital admissions [12]. This study provisionally supports this, showing static hospitalisation rates in the i-CGA group compared to substantially increased control group rates. With a larger sample, there may be a statistically significant effect. As with another recent study [5], this study found high adherence to end-of-life preferences. As i-CGA increased ACP documentation, it seems plausible hospital admissions would decrease in accordance with residents’ documented wishes.

The significant mortality difference for those with severe frailty was interesting. A German study of community-based proactive CGA for frail older patients living in their own homes resulted in a 20% mortality reduction [25]. There is little data published on proactive CGAs in care homes [26], but plausible survival benefit may be conferred by the reduced hospitalisation rates stemming from ACP preferences, and/or relatively rapidly reduced morbidity resulting from CGA e.g. in identifying medical issues or deprescribing (thus reducing falls and physical/cognitive frailty severity) [27]. Although the different follow-up timing for the intervention group could mean the pandemic may have contributed to the slightly increased mortality in those with mild/moderate frailty, it is also plausible that the relatively reduced admission rates for those with severe frailty (following ACPs) during the pandemic was protective.

### Implications for research and practice

Our proactive primary care-led intervention of i-CGA and ACP for adults with severe frailty in care homes seems to have conferred significant benefits, including apparently reduced mortality and high adherence to expressed ACP preferences, adding to the growing evidence that proactive CGA and ACP is beneficial for people who have received a frailty diagnosis. Our process enables, for the first time, proactive and systematic CGA and ACP completion in primary care by General Practitioners. Although only two practitioners completed i-CGAs in this study, its perceived usefulness and ease of use means it has now been taken up and is used by many more practitioners. This is particularly relevant for the future of general practice given the proportionate decrease in specialist geriatricians and ageing population/increased demand for geriatric medicine. Further analysis and implementation of i-CGA with individuals living in different settings will be enlightening, and could provide insight on the role of ACP compared to i-CGA in reducing mortality. We propose a multi-site wedged-design study using i-CGA over a longer period, with larger numbers of residents as well as people living with frailty in their own homes. Future analyses should include patient-reported outcome measures, out-of-hours consultations, ambulance interactions, falls, fractures, and economic evaluation.

## Conclusion

A proactive, IT-assisted, Comprehensive Geriatric Assessment and Advance Care Planning process in residential care homes may offer significant benefits to residents with severe frailty, including a significant survival advantage and significantly increased documentation of end-of-life preferences compared to usual care. Further research is needed to investigate whether i-CGA can also reduce unplanned hospitalisations. The authors propose a multi-site i-CGA study, involving more care home residents and people in their own homes.

### Electronic supplementary material

Below is the link to the electronic supplementary material.

Supplementary Material 1

## Data Availability

The datasets used and analysed during the current study are currently not publicly available. However, these can be obtained from the corresponding author on reasonable request.

## Abbreviations

ACP: Advanced Care Planning / Advanced Care Plan
CGA: Comprehensive Geriatric Assessment
COVID-19: Coronavirus Disease
e-TEP: Electronic Treatment and Escalation Plan
GP: General Practitioner
i-CGA: IT-assisted Comprehensive Geriatric Assessment
THS: Target Health Solutions, a company that enhances primary care IT
