# Supplementary material for: IT-assisted comprehensive geriatric assessment for residents in care homes: quasi-experimental longitudinal study
Source: BMC Geriatr. 2024 Mar 19;24:269. doi: 10.1186/s12877-024-04824-6 (PMC10949740; doi:10.1186/s12877-024-04824-6)
Supplement: Supplementary file 1 — Supplementary Material 1 [file 12877_2024_4824_MOESM1_ESM.docx]

**IT-assisted comprehensive geriatric assessment for care home residents: a quasi-experimental longitudinal study**

**Supplementary Tables**

**Supplementary Table 1 – Advance care planning preferences and mortality including place of death. This shows complete data including for those with mild and moderate frailty.**

| **Advance care planning, CPR and hospitalisation preferences** | **Number of residents, n (%)** | | **Number of deaths,**  **n (%)** | | **Place of death** | | | | | |
| --- | --- | --- | --- | --- | --- | --- | --- | --- | --- | --- |
|  |  |  |  |  | **Died in care home,**  **n (%)** | | **Died in hospital, n (%)** | | **Died in unknown location, n (%)** | |
| **Control group** | | | | | | | | | | |
| **Overall** | 100 |  | 57 | (57) | 39 | (68) | 13 | (23) | 5 | (9) |
| Advance care plan in place | 72 | (72) |  |  |  |  |  |  |  |  |
| Prefers natural death (DNAR) | 70 | (70) |  |  |  |  |  |  |  |  |
| Prefers CPR | 1 | (1) |  |  |  |  |  |  |  |  |
| Prefers to remain undecided | 1 | (1) |  |  |  |  |  |  |  |  |
| Prefers to be hospitalised if more unwell | 25 | (25) | 6 | (24) | 2 | (33) | 3 | (50) | 1 | (17) |
| Prefers not to be hospitalised if more unwell | 46 | (46) | 26 | (57) | 23 | (88) | 2 | (8) | 1 | (4) |
| No advance care plan/not stated | 29 | (29) | 25 | (86) | 14 | (56) | 8 | (32) | 3 | (12) |
| **Mild frailty** | 13 |  | 3 | (23) | 1 | (33) | 2 | (67) | 0 | (0) |
| Prefers to be hospitalised if more unwell | 4 | (31) | 0 | (0) | 0 | (0) | 0 | (0) | 0 | (0) |
| Prefers not to be hospitalised if more unwell | 5 | (38) | 0 | (0) | 0 | (0) | 0 | (0) | 0 | (0) |
| No advance care plan/not stated | 4 | (31) | 3 | (75) | 1 | (33) | 2 | (67) | 0 | (0) |
| **Moderate frailty** | 31 |  | 11 | (35) | 7 | (64) | 2 | (18) | 2 | (18) |
| Prefers to be hospitalised if more unwell | 6 | (19) | 0 | (0) | 0 | (0) | 0 | (0) | 0 | (0) |
| Prefers not to be hospitalised if more unwell | 16 | (52) | 5 | (31) | 5 | (100) | 0 | (0) | 0 | (0) |
| No advance care plan/not stated | 9 | (56) | 6 | (67) | 2 | (33) | 2 | (33) | 2 | (33) |
| **Severe frailty** | 56 |  | 43 | (77) | 31 | (72) | 9 | (21) | 3 | (7) |
| Prefers to be hospitalised if more unwell | 15 | (27) | 6 | (40) | 2 | (33) | 3 | (50) | 1 | (17) |
| Prefers not to be hospitalised if more unwell | 25 | (45) | 21 | (84) | 18 | (86) | 2 | (10) | 1 | (5) |
| No advance care plan/not stated | 16 | (29) | 16 | (100) | 11 | (69) | 4 | (25) | 1 | (6) |
| **i-CGA group** | | | | | | | | | | |
| **Overall** | 196 |  | 94 | (48) | 73 | (78) | 15 | (16) | 6 | (6) |
| Advance care plan in place | 196 | (100) |  |  |  |  |  |  |  |  |
| Prefers natural death (DNAR) | 191 | (97) |  |  |  |  |  |  |  |  |
| Prefers CPR | 5 | (3) |  |  |  |  |  |  |  |  |
| Prefers to remain undecided | 0 | (0) |  |  |  |  |  |  |  |  |
| Prefers to be hospitalised if more unwell | 51 | (26) | 22 | (43) | 8 | (36) | 12 | (55) | 2 | (9) |
| Prefers not to be hospitalised if more unwell | 145 | (74) | 72 | (50) | 65 | (90) | 3 | (4) | 4 | (6) |
| No advance care plan/not stated | 0 | (0) | 0 | (0) | 0 | (0) | 0 | (0) | 0 | (0) |
| **Mild frailty** | 16 |  | 5 | (31) | 3 | (60) | 2 | (40) | 0 | (0) |
| Prefers to be hospitalised if more unwell | 9 | (56) | 3 | (33) | 1 | (33) | 2 | (67) | 0 | (0) |
| Prefers not to be hospitalised if more unwell | 7 | (44) | 2 | (29) | 2 | (100) | 0 | (0) | 0 | (0) |
| No advance care plan/not stated | 0 | (0) | 0 | (0) | 0 | (0) | 0 | (0) | 0 | (0) |
| **Moderate frailty** | 69 |  | 28 | (41) | 19 | (68) | 9 | (32) | 0 | (0) |
| Prefers to be hospitalised if more unwell | 25 | (36) | 10 | (40) | 3 | (30) | 7 | (70) | 0 | (0) |
| Prefers not to be hospitalised if more unwell | 44 | (64) | 18 | (41) | 16 | (89) | 2 | (11) | 0 | (0) |
| No advance care plan/not stated | 0 | (0) | 0 | (0) | 0 | (0) | 0 | (0) | 0 | (0) |
| **Severe frailty** | 111 |  | 61 | (55) | 51 | (84) | 4 | (7) | 6 | (10) |
| Prefers to be hospitalised if more unwell | 17 | (15) | 9 | (53) | 4 | (44) | 3 | (33) | 2 | (22) |
| Prefers not to be hospitalised if more unwell | 94 | (85) | 52 | (55) | 47 | (90) | 1 | (2) | 4 | (8) |
| No advance care plan/not stated | 0 | (0) | 0 | (0) | 0 | (0) | 0 | (0) | 0 | (0) |

**Supplementary Table 2 – Means and 95% confidence intervals of the number of unplanned hospital admissions/person year alive, and the unplanned hospital bed days/person year alive in the control and intervention groups (IT-assisted comprehensive geriatric assessment, i-CGA), for the year before and after, and the overall treatment difference. This shows complete data including for those with mild and moderate frailty.**

|  | **Control** | | | **i-CGA** | | | **Treatment difference** | |
| --- | --- | --- | --- | --- | --- | --- | --- | --- |
|  | **Before** | **After** | **Difference** | **Before** | **After** | **Difference** | **Difference** | **p-value** |
| **Unplanned admissions** | | | | | | | | |
| All residents | 0.85  ±0.20 | 1.86  ±0.84 | 1.01  ±0.88 | 0.83  ±0.17 | 0.87  ±0.29 | 0.04  ± 0.34 | -0.97 ± 0.94 | 0.16 |
| Mild  frailty | 0.54  ±0.61 | 2.24  ±1.63 | 1.70  ±1.93 | 0.25  ±0.22 | 0.84  ±0.93 | 0.59  ± 1.05 | -1.11 ± 2.12 | 0.16 |
| Moderate frailty | 0.93  ±0.38 | 1.36  ±0.88 | 0.42  ±0.87 | 0.94  ±0.30 | 1.17  ±0.53 | 0.23  ±0.60 | -0.19 ± 1.04 | 0.99 |
| Severe frailty | 0.87  ±0.25 | 2.05  ±1.37 | 1.18  ±1.46 | 0.84  ±0.24 | 0.68  ±0.37 | -0.16  ±0.10 | -1.34 ± 1.52 | 0.22 |
| **Unplanned bed days** | | | | | | | | |
| All residents | 8.8  ±2.9 | 17.2  ±8.1 | +8.4  ±8.0 | 7.1  ±1.6 | 8.7  ±4.0 | +1.7  ± 4.1 | -6.8 ± 9.0 | 0.45 |
| Mild  frailty | 4.0  ±6.6 | 29.6  ±28.9 | +25.6  ±33.1 | 2.4  ±2.8 | 8.5  ±9.3 | +6.1  ±11.1 | -19.5 ± 34.3 | 0.16 |
| Moderate frailty | 8.6  ±4.5 | 16.7  ±15.9 | +8.2  ±15.2 | 8.1  ±3.0 | 11.4  ±6.8 | +3.2  ±6.5 | -4.9 ± 16.4 | 0.84 |
| Severe frailty | 10.1  ±4.2 | 14.6  ±9.4 | +4.6  ±9.6 | 7.1  ±2.2 | 7.1  ±5.4 | +0.04  ±5.9 | -4.5 ± 11.2 | 0.99 |

**Supplementary Figure 1: Mean number of unplanned bed days/person year alive overall in the control group (baseline and follow up year) and the intervention group (baseline and year following IT-assisted comprehensive geriatric assessment, i-CGA). Graphs shown for overall, and by frailty status**
